# Grape-Seed Proanthocyanidin Extract as Suppressors of Bone Destruction in Inflammatory Autoimmune Arthritis

**DOI:** 10.1371/journal.pone.0051377

**Published:** 2012-12-10

**Authors:** Jin-Sil Park, Mi-Kyung Park, Hye-Joa Oh, Yun-Ju Woo, Mi-Ae Lim, Jong-Ho Lee, Ji Hyeon Ju, Young Ok Jung, Zang Hee Lee, Sung-Hwan Park, Ho-Youn Kim, Mi-La Cho, Jun-Ki Min

**Affiliations:** 1 The Rheumatism Research Center, Catholic Research Institute of Medical Science, The Catholic University of Korea, Seoul, Republic of Korea; 2 Division of Rheumatology, Department of Internal Medicine, Hallym University Kang-Nam Sacred Heart Hospital, Seoul, Republic of Korea; 3 Department of Cell and Developmental Biology, School of Dentistry, Seoul National University, Seoul, Republic of Korea; 4 Immune Tolerance Research Center, Convergent Research Consortium for Immunologic Disease (CRCID); The Catholic University of Korea College of Medicine, Seoul, Republic of Korea; Faculté de médecine de Nantes, France

## Abstract

Chronic autoimmune inflammation, which is commonly observed in rheumatoid arthritis (RA), disrupts the delicate balance between bone resorption and formation causing thedestruction of the bone and joints. We undertook this study to verify the effects of natural grape-seed proanthocyanidin extract (GSPE), an antioxidant, on chronic inflammation and bone destruction. GSPE administration ameliorated the arthritic symptoms of collagen-induced arthritis (CIA), which are representative of cartilage and bone destruction. GSPE treatment reduced the formation of tartrate-resistant acid phosphatase (TRAP)-positive multinucleated cells and osteoclast activity and increased differentiation of mature osteoblasts. Receptor activator of NFκB ligand expression in fibroblasts from RA patients was abrogated with GSPE treatment. GSPE blocked human peripheral blood mononuclear cell-derived osteoclastogenesis and acted as an antioxidant. GSPE improved the arthritic manifestations of CIA mice by simultaneously suppressing osteoclast differentiation and promoting osteoblast differentiation. Our results suggest that GSPE may be beneficial for the treatment of inflammation-associated bone destruction.

## Introduction

Rheumatoid arthritis (RA) is a systemic inflammatory disease characterized by hyperplasia of synovial tissue and progressive destruction of joint structure. Osteoporosis and related fragility fractures severely affect the quality of life of patients with RA. In pathological conditions of RA, osteoclasts invade the juxta-synovial bone and cause the formation of pannus [1]. Increased osteoclast activity can cause severe damage to the bone, leading to the progressive destruction of arthritic joints [2]. Osteoclasts are multinucleated cells that exhibit tartrate-resistant acid phosphatase (TRAP) activity and have the ability to form resorption pits in bone. Osteoclast formation is a contact-dependent process that is controlled by mesenchymal cells, such as osteoblasts and fibroblasts, which provide signals that are essential for its differentiation [3]. Osteoclasts differentiate from mononuclear cells of the monocyte/macrophage lineage upon stimulation by two integral factors: the monocyte/macrophage colony–stimulating factor (M-CSF) via its cognate receptor c-fms, which is expressed in osteoclast progenitor cells and the novel tumor necrosis factor (TNF) ligand member termed receptor activator of nuclear factor kappa-B ligand (RANKL; also termed TRANCE/ODF/OPGL) [3], [4], [5].

Bone homeostasis is maintained by the balance between two major bone remodeling processes: bone resorption by osteoclasts and bone formation by osteoblasts [6], [7]. Osteoblasts play a central role in bone formation by synthesizing multiple bone matrix proteins and regulating osteoclast maturation by producing soluble factors and cognate interactions, causing bone resorption [8]. Chronic, systemic inflammatory disorders, such as RA, causes an imbalance between bone formation and destruction by cytokines and matrix-degrading enzymes produced by effector cells, including osteoclasts, fibroblasts, leukocytes, and chondrocytes [9].

Previous studies have shown that there are an abundant of osteoclasts at the bone–synovium interface in the joints of RA patients [4]. RANKL is highly expressed in synovial fibroblasts of arthritic joints and is responsible for the abnormal activation of osteoclasts [10]. Several antiresorptive therapies (i.e., osteoprotegerin, anti-RANKL antibody, and anti-TNF-α antibody) were shown to ameliorate bone damage in models of inflammatory bone destruction [2], [11], [12], [13]. In addition to these therapeutic approaches, a therapeutic approach that promotes bone formation is considered an optimal adjunctive treatment modality.

Grape-seed proanthocyanidin extract (GSPE), a naturally occurring polyphenolic compound from the seeds of *Vitis vinifera*, possesses a wide range of biological properties against oxidative stress [14], [15], [16]. GSPE comprises a combination of ingredients: 15% (+)-catechin and (–)-epicatechin; 80% (–) epicatechin 3-*O*-gallate, dimers, trimers, tetramers, and their gallates; and 5% pentamers, hexamers, heptamers, and their gallates [17]. Its active constituents are proanthocyanidins, which can scavenge free radicals required for calcium absorption [18]. The relationship between other flavonoids, such as genistein (4′,5,7-trihydroxyisoflavone) and daidzein (4′,7-dihydroxyisoflavone), and bone health has been reported [19], [20]. Furthermore, several reports have shown that reactive oxygen species (ROS) are responsible for tissue damage and inflammation in many diseases, including atherosclerosis, cancer, myocardial infarction, and muscle disease [21]. Thus, GSPE may play a role in the regulation of bone homeostasis.

In this study, the effects of GSPE on inflammation- and oxygen radical-associated bone destruction were investigated. GSPE improved the arthritic manifestations of collagen-induced arthritis (CIA) in mice. GSPE simultaneously suppressed osteoclast differentiation and promoted osteoblast differentiation. GSPE may be a beneficial adjunctive modality in the treatment of inflammation-associated bone destruction, such as RA.

**Figure 1:**
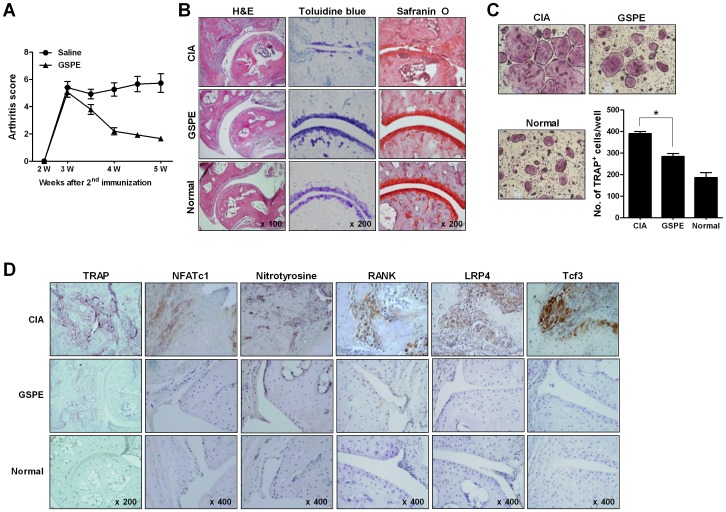
*In vivo* effects of GSPE treatment. GSPE (100 mg/kg) or saline was administered via intraperitoneal injection twice weekly after booster immunization over the course of 18 days. (A) The severity of arthritis was compared. (B) Sections of articular tissue from saline-treated CIA, GSPE-treated CIA, and normal mice were stained with H&E, safranin O, and toluidine blue. (C) BMC-derived preosteoclasts from saline-treated CIA mice, GSPE-treated mice, and normal mice were cultured in the presence of M-CSF (10 ng/mL) and RANKL (100 ng/mL) for four days (original magnification, ×100). Cells were fixed and stained for TRAP. TRAP^+^ MNCs were counted using light microscopy. Values represent mean ± SD. *P<0.05 versus saline-treated CIA mice. (D) Representative histological features of the tibiotalar joint of saline-treated CIA mice, GSPE-treated mice, and normal mice. TRAP staining and immunohistochemical staining for NFATc1, nitrotyrosine, RANK, LRP4, and Tcf3 are shown.

## Materials and Methods

### Animals

Four- to six-week-old male DBA/1J mice were purchased from SLC, Inc. (Shizuoka, Japan). Mice were maintained under specific pathogen-free conditions at the Institute of Medical Science at the Catholic University of Korea and were provided standard mouse chow (Ralston Purina, St. Louis, MO, USA) and water *ad libitum*. All experimental procedures were examined and approved by the Animal Research Ethics Committee of the Catholic University of Korea (permit number: CUMC-20), which conforms to all National Institutes of Health of the USA guidelines. All surgeries were performed under isoflurane anesthesia, and all efforts were made to minimize suffering.

**Figure 2:**
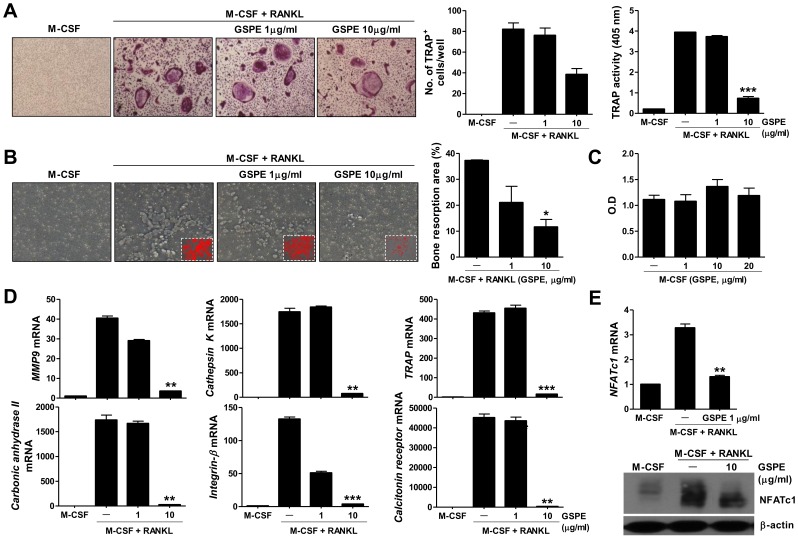
Suppressive effects of GSPE on osteoclast differentiation *in vitro*. (A) Mouse preosteoclasts from BMMs were incubated for four days in medium containing M-CSF or M-CSF and RANKL with or without various concentrations of GSPE (1 or 10 µg/mL). Cells were fixed and stained for TRAP (original magnification, ×100). The number of TRAP^+^ cells (nuclei >10) was counted using light microscopy. The TRAP activity of the cells was determined using a TRACP & ALP assay kit. (B) Osteoclasts were generated in 48-well OAAS plates in the presence or absence of GSPE for six days to assess their functional activity using a resorption assay system (original magnification, ×100). Resorbed areas (red) were examined using the Tomoro analySIS TS Lite program. Data represent the average values of three independent experiments. (C) Mouse preosteoclasts were cultured with GSPE for 4 days and then reacted with CCK-8 (10 µL) for 2 h. The absorbance was measured at 450 nm. (D) Mouse preosteoclasts were incubated for four days in medium containing M-CSF or M-CSF and RANKL with or without various concentrations of GSPE. RNA was then extracted and the expression of osteoclast-related genes was quantified using real-time PCR. mRNA levels were normalized to β-actin levels. Data represent the average values of three independent experiments. (E) The mRNA expression of NFATc1 was measured using real-time PCR (upper). Data represent the average values of three independent experiments. **P<0.01, as compared with the M-CSF plus RANKL condition. Osteoclasts were generated in the presence or absence of GSPE for 24 h, and immunoblot analysis of NFATc1 was performed (lower). *P<0.05, **P<0.01, and ***P<0.001, as compared with the M-CSF plus RANKL condition.

### GSPE Extraction

The bark, seeds and branches of *Vitis vinifera* were compressed, washed with water, and dried in a rotating oven, and the seeds of *V. vinifera* were isolated. Approximately 1 kg of *V. vinifera* seeds were pulverized and subjected to extraction using 500 mL of an acetone/water solution (acetone/water = 8/2, v/v). The extraction was repeated three times, and the extract was then gathered and filtered. The filtrate was concentrated under reduced pressure to remove acetone and then filtered again. The filtrate was subjected to extraction three times using 250 mL ethyl acetate and dehydrated using anhydrous sodium sulfate. The extract was concentrated under reduced pressure to remove ethyl acetate, the concentrate was dissolved in 500 mL water, and the solution was spray-dried to obtain about 20 g extract powder (first extract). The first extract and the second extract were mixed to obtain about 35 g *V. vinifera* pip extract. The obtained extract was hydrolyzed by heating in a diluted acid solution, and the procyanidolic value was measured by quantifying procyanidolic oligomers. As a result, the procyanidolic value was about 98%. In addition, the amount of proanthocyanidin was 98.5%. Thus, the extract included a large amount of oligomers of which at least two monomers, such as (+) catechin and (−) epicatechin, were polymerized.

**Figure 3:**
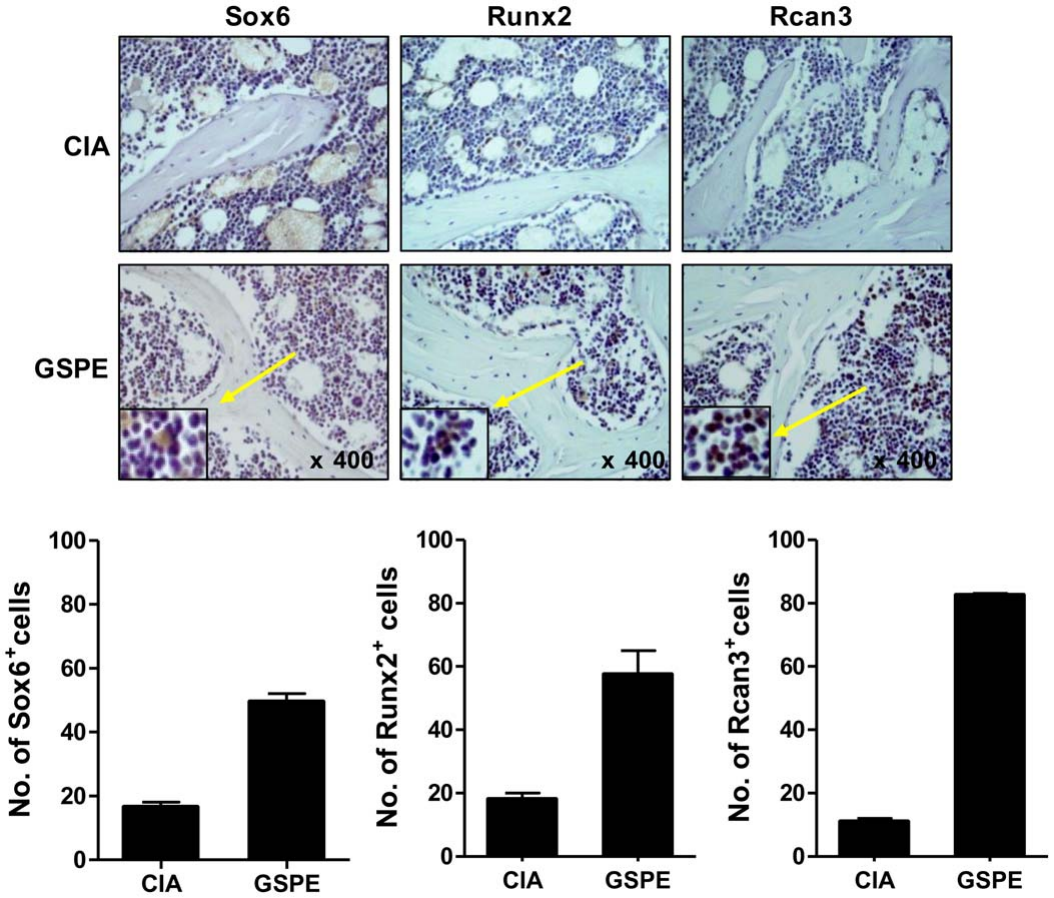
Expression of Sox6, Runx2, and Rcan3 in inflamed joints. Representative histological features of the tibiotalar joints of saline-treated CIA mice and GSPE-treated mice. Immunohistochemical staining for Sox6, Runx2, and Rcan3 is shown. Graphs represent the quantified degree of Sox6, Runx2 and Rcan3 expressed cells, respectively.

**Figure 4:**
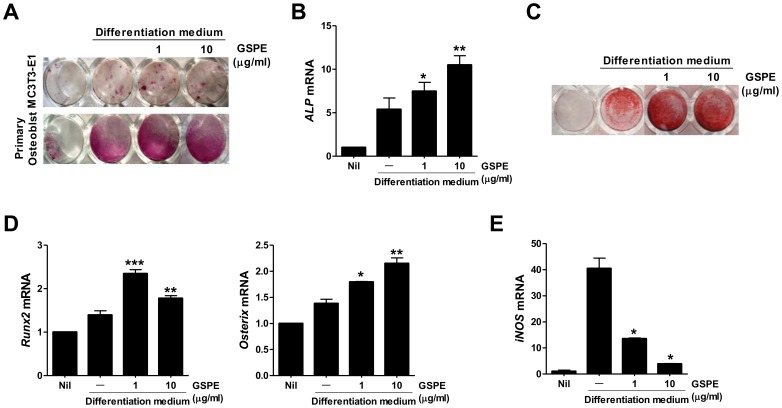
Increased osteoblast response after GSPE treatment. (A) Primary osteoblasts and MC3T3-E1 cell lines were cultured for three and seven days, respectively, in the presence of 10 mM β-glycerophosphate, 50 µg/mL ascorbic acid, and 10 ng/mL rhBMP2 (differentiation medium) with or without GSPE to induce osteoblast differentiation. Osteoblast differentiation was then evaluated using ALP staining. (B) Primary osteoblasts were cultured for three days, and RNA was extracted. Real-Time PCR was performed for ALP. Data represent the average values of three independent experiments. (C) Primary osteoblasts were cultured for 14 days in the presence of 10 mM β-glycerophosphate, 50 µg/mL ascorbic acid, and 10 ng/mL rhBMP2 with or without GSPE to induce osteoblast differentiation. Osteoblast differentiation was evaluated using alizarin red O staining in primary osteoblasts. (D) Primary osteoblasts were cultured for six days and RNA was extracted. Real-Time PCR was performed to detect Runx2 and osterix expression. Data represent the average values of three independent experiments. (E) Expression of iNOS was detected in 6-day cultured primary osteoblasts by real-time PCR. *P<0.05, **P<0.01, and ***P<0.001, as compared with the differentiation medium condition.

### Induction of CIA and Treatment with GSPE

To induce CIA in mice, 0.1 mL of an emulsion containing 100 µg bovine type II collagen (CII) and complete Freund’s adjuvant (Chondrex, Redmond, WA, USA) was injected intradermally into the base of the tail as the primary immunization. Two weeks later, 100 µg CII, dissolved and emulsified 1∶1 with incomplete Freund’s adjuvant (Difco, Detroit, MI, USA), was administered into the footpad as a booster injection. To assess the influence of GSPE on symptom severity in the CIA model, mice were treated with 100 mg/kg GSPE in saline or with vehicle alone via intraperitoneal injections twice weekly after booster immunization over the course of 18 days. Untreated normal mice were used as controls. The severity of arthritis was recorded using the mean arthritis index on a scale of 0–4, as previously described [22]. The final value represents the average recorded by three independent observers.

**Figure 5:**
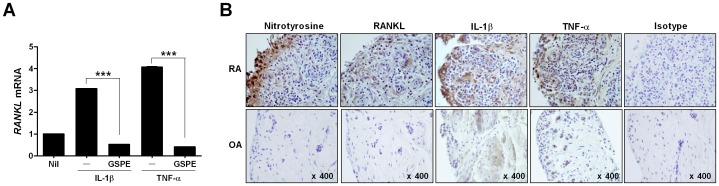
Effect of GSPE on RANKL mRNA expression in RA synovial fibroblasts. (A) RA synovial fibroblasts were cultured for 72 h with 1 ng/mL TNF-α or 0.1 ng/mL IL-1β in the presence of 10 µg/mL GSPE. Analysis of RANKL mRNA expression was performed using Real-Time PCR. Values represent the mean ± SD for three independent experiments. ***P<0.001 versus the IL-1β- or TNF-α-stimulated condition. (B) Immunohistochemical detection of nitrotyrosine, RANKL, IL-1β, and TNF-α in the synovium of patients with RA or OA. Background immunohistochemical staining of synovium tissue was performed with anti-isotype antibodies. All tissues were counterstained with hematoxylin (original magnification, ×400).

### Immunohistochemistry

Mouse joint tissues were fixed in 4% paraformaldehyde, decalcified in EDTA bone decalcifier, embedded in paraffin, and sectioned [23]. The sections were stained with hematoxylin and eosin (H&E), safranin O, and toluidine blue to detect proteoglycans. The sections were de-waxed using xylene and dehydrated in a gradient of alcohols. Endogenous peroxidase activity was quenched with 3% hydrogen peroxide (H_2_O_2_) in methanol. Immunohistochemistry was performed using the Vectastain ABC kit (Vector Laboratories, Burlingame, CA, USA). Tissues were first incubated with primary antibodies to nitrotyrosine, nuclear factor of activated T cell cytoplasmic 1 (NFATc1), RANK, LDL receptor-related protein 4 (LRP4), T cell factor 3 (Tcf3), Sox6, Runt-related transcription factor 2 (Runx2), and regulators of calcineurin 3 (Rcan3) (Santa Cruz Biotechnology, Santa Cruz, CA, USA) overnight at 4°C. The tissues were then incubated with a biotinylated secondary linking antibody and a streptavidin–peroxidase complex for 1 h. The final color product was developed using DAB chromogen (DAKO, Carpinteria, CA, USA). The sections were counterstained with hematoxylin. Samples were photographed with an Olympus photomicroscope (Tokyo, Japan).

**Figure 6:**
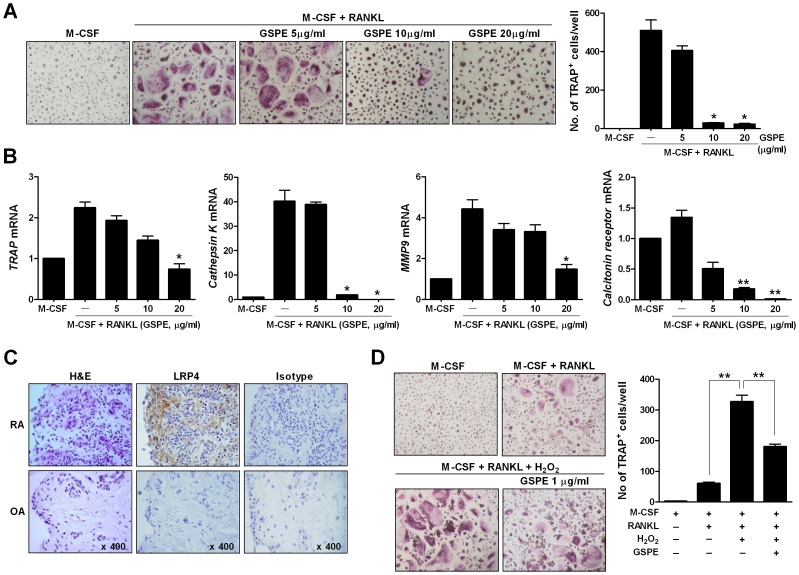
Suppressive effects of GSPE on human monocyte-derived osteoclasts. (A) Monocytes from human PBMC were cultured for three days with 100 ng/mL M-CSF to form macrophage-like preosteoclasts. After three days, preosteoclasts were cultured for an additional nine days in the presence of 25 ng/mL M-CSF, 30 ng/mL RANKL, and various concentrations of GSPE to generate osteoclasts. Cells were fixed and stained for TRAP (original magnification, ×100). The number of TRAP^+^ MNCs (nuclei >10) was counted using light microscopy. Data represent the average values of three independent experiments. (B) The expression of TRAP, cathepsin K, MMP9, and calcitonin receptor mRNA was quantified using real-time PCR. mRNA levels were normalized to β-actin levels. Data represent the average values of three independent experiments. (C) H&E staining and immunostaining for LRP4 are shown in the synovium of patients with RA or OA. (D) Monocytes from human PBMC were cultured for three days with 100 ng/mL M-CSF to form preosteoclasts. After three days, preosteoclasts were cultured for an additional nine days in the presence of 25 ng/mL M-CSF, 30 ng/mL RANKL, and with or without GSPE and/or H_2_O_2_. Cells were fixed and stained for TRAP (original magnification, ×100). The number of TRAP^+^ MNCs (nuclei >10) was counted using light microscopy. Data represent the average values of three independent experiments. *P<0.05 and **P<0.01, as compared with the M-CSF plus RANKL condition.

### Mouse *in vitro* Osteoclastogenesis

Bone marrow-derived monocyte/macrophages (BMMs) were isolated from the tibias and femurs of mice by flushing the bone marrow cavity with minimum essential medium-α (Invitrogen, Carlsbad, CA, USA). The cells were allowed to incubate for 12 h to separate nonadherent and adherent cells. Nonadherent cells were seeded in 48-well plates at 1×10^5^ cells/well and cultured in the presence of 10 ng/mL rh M-CSF (R&D Systems) for three days to form macrophage-like osteoclast precursor cells (preosteoclasts). Three days later, the nonadherent cells were washed out and preosteoclasts were cultured further in the presence of 10 ng/mL M-CSF, 100 ng/mL RANKL (Peprotech, London, UK), and various concentrations of GSPE for four days to generate osteoclasts. On day 2, the medium was replaced with fresh medium containing M-CSF, RANKL, and GSPE.

### Human *in vitro* Osteoclastogenesis

Peripheral blood mononuclear cells (PBMCs) obtained from normal healthy volunteers were separated from buffy coats using Ficoll–Hypaque (Pharmacia Biotech, Piscataway, NJ). Approval by the ethics committee of the Seoul St. Mary’s Hospital (Seoul, Korea) was obtained for all procedures and informed consent was obtained from all healthy volunteers. Red blood cells were removed, and the cells were seeded into 24-well plates at 5×10^5^ cells/well and incubated at 37°C for 2 h to separate the nonadherent and adherent cells. The adherent cells were washed with PBS (Gibco, Burlingame, CA, USA) and cultured with 100 ng/mL M-CSF for three days. After three days, these preosteoclasts were cultured further in the presence of 25 ng/mL M-CSF, 30 ng/mL RANKL, and various concentrations of GSPE for nine days to generate osteoclasts. On day 3, the medium was replaced with fresh medium containing M-CSF, RANKL, and GSPE.

### Bone Resorption Analysis

Mouse BMMs were prepared using the method described above and were cultured in 48-well OAAS plates (Osteogenic Core Technologies, Choongnam, Korea). Erosive areas were identified using the Tomoro analySIS TS Lite program (Olympus, Münster, Germany).

### TRAP Staining

A commercial TRAP kit (Sigma, St Louis, MO, USA) was used according to the manufacturer’s instructions; however, counterstaining with hematoxylin was omitted. TRAP-positive multinucleated cells (MNCs) containing three or more nuclei were counted as osteoclasts.

### TRAP Activity

TRAP activity was measured using the TRACP & alkaline phosphatase (ALP) assay kit (Takara, Shiga, Japan) according to the manufacturer’s instructions.

### Cell Viability Analysis

Cell viability was determined using the CCK-8 kit (Dojindo Laboratories) according to the manufacturers’ instructions. Briefly, preosteoclasts were cultured with GSPE for 4 days, CCK-8 (10 µL/well) was added and then incubated for 2 h. The absorbance was measured at 450 nm on a microplate reader.

### Culture of Primary Mouse Osteoblastic Cells

Calvarias were isolated from one-day-old neonatal Crl:CD1 mice and digested with 0.1% collagenase (Sigma) and 0.2% dispase (Roche, Indianapolis, IN, USA) for 5 min at 37°C. After removal of the medium, the remaining calvarias were digested four times for 10 min at 37°C. Cell fractions were collected and used as primary murine osteoblasts [24]. Cells were cultured for three days, and adherent cells were used as osteoblasts. Primary murine osteoblasts and MC3T3-E1 murine osteoblastic cell lines (kindly provided by Dr. ZH Lee, Seoul National University, Korea) were plated at 5×10^4^ cells/well onto 48-well plates [25]. After 24 h, cells were cultured in the presence of 10 mM β-glycerophosphate (Sigma), 50 µg/mL ascorbic acid (Sigma), and 10 or 50 ng/mL recombinant human bone morphogenetic protein 2 (rhBMP2) (Peprotech, Rocky Hill, NJ, USA) with or without GSPE to induce osteoblast differentiation. On day 3, the medium was replaced with fresh medium containing β-glycerophosphate, ascorbic acid, and rhBMP2 with or without GSPE.

### ALP and Alizarin Red O Staining

Cells were stained for ALP according to the manufacturer’s instructions (Sigma). For alizarin red O (Sigma) staining, cells were fixed in 10% formalin in sterile PBS and stained with 2% alizarin red O solution.

### Gene Expression Analysis Using Real-time PCR

PCR amplification and analysis were performed on a LightCycler 2.0 instrument (Roche Diagnostic, Mannheim, Germany) with software version 4.0. All reactions were performed using LightCycler FastStart DNAmaster SYBR green I (Takara), according to the manufacturer’s instructions. The following primers were used for mouse sequences: matrix metalloproteinase 9 (MMP9), 5′–CTGTCCAGACCAAGGGTACAGCCT–3′ (sense), 5′–GAGGTATAGTGGGACACATAGTGG–3′ (antisense); cathepsin K, 5′–CAGCAGAGGTGTGTACTATG–3′ (sense), 5′–GCGTTGTTCTTATTCCGAGC–3′ (antisense); TRAP, 5′–TCCTGGCTCAAAAAGCAGTT–3′ (sense), 5′–ACATAGCCCACACCGTTCTC–3′ (antisense); carbonic anhydrase II, 5′– TGGTTCACTGGAACACCAAA–3′ (sense), 5′– AGCAAGGGTCGAAGTTAGCA –3′ (antisense); integrin-β, 5′–CTGTGGGCTTTAAGGACAGC–3′ (sense), 5′–GAGGGTCGGTAATCCTCCTC–3′ (antisense); calcitonin receptor, 5′–CGGACTTTGACACAGCAGAA–3′ (sense), 5′–AGCAGCAATCGACAAGGAGT–3′ (antisense); NFATc1, 5′–CGGGAAGAAGATGGTGCTGT–3′ (sense), 5′–TTGGACGGGGCTGGTTAT–3′ (antisense); and β-actin, 5′–GTACGACCAGAGGCATACAGG–3′ (sense), 5′–GATGACGATATCGCTGCGCTG–3′ (antisense); ALP, 5′–CCTGACTGTGGTTACTGCTG–3′ (sense), 5′–GAGCGTAATCTACCATGGAG–3′ (antisense); Runx2, 5′–TCTGGCCTTCCACTCTCAGT–3′ (sense), 5′–TATGGAGTGCTGCTGGTCTG–3′ (antisense); osterix, 5′–TGATGATAATTTATTGCCCC–3′ (sense), 5′–CTGACCCGTCATCATAACTT–3′ (antisense); inducible nitric oxide synthase (iNOS), 5′–CAGCTGGGCTGTACAAACCTT–3′ (sense), 5′–CATTGGAAGTGAAGCGTTTCG–3′ (antisense); and GAPDH, 5′–CATGGCCTTCCGTGTTCCTA–3′ (sense), 5′–CCTGCTTCACCACCTTCTTGAT–3′ (antisense). The following primers were used for human sequences: RANKL, 5′–ACCAGCATCAAAATCCCAAG–3′ (sense), 5′–CCCCAAAGTATGTTGCATCC–3′ (antisense); TRAP, 5′–GACCACCTTGGCAATGTCTCTG–3′ (sense), 5′–TGGCTGAGGAAGTCATCTGAGTTG–3′ (antisense); cathepsin K, 5′–TGAGGCTTCTCTTGGTGTCCATAC–3′ (sense), 5′–AAAGGGTGTCATTACTGCGGG–3′ (antisense); MMP9, 5′–CGCAGACATCGTCATCCAGT–3′ (sense), 5′–GGATTGGCCTTGGAAGATGA–3′ (antisense); calcitonin receptor, 5′–TGGTGCCAACCACTATCCATGC–3′ (sense), 5′–CACAAGTGCCGCCATGACAG–3′ (antisense); and β-actin, 5′–GGACTTCGAGCAAGAGATGG–3′ (sense), 5′–TGTGTTGGCGTACAGGTCTTTG–3′ (antisense). The level of mRNA expression was normalized to β-actin or GAPDH expression levels.

### RANKL Production by RA Synovial Fibroblasts

All RA patients met the American College of Rheumatology 1987 revised criteria for the classification of RA [26]. Fibroblast-like synoviocytes (FLS) from RA patients were obtained as described previously [27]. Fibroblasts from RA patients at passage 4–8 were seeded onto 60-mm dishes at 1×10^5^ cells/dish in Dulbecco’s modified Eagle’s medium containing 10% FBS overnight to attach the cells to the wells, and the cells were serum-starved with 1× insulin–transferrin–selenium A (Invitrogen, Carlsbad, CA, USA) for 12 h. After a PBS washout, cells were stimulated with 1 ng/mL TNF-α or 0.1 ng/mL interleukin (IL)-1β in the presence or absence of 10 µg/mL GSPE for 72 h, and RANKL mRNA expression was measured.

### Immunoblot Analysis

BMMs were cultured with M-CSF and RANKL in the presence or absence of GSPE for 24 h. Cells were then harvested and lysed with lysis buffer. Protein concentration was measured using the Bradford method (Bio-Rad, Hercules, CA, USA). Protein samples were separated using 10% SDS-PAGE and transferred onto nitrocellulose membranes (Amersham Pharmacia Biotech, Piscataway, NJ, USA). For Western blot hybridization, the membrane was preincubated with blocking buffer for 2 h, and then incubated with primary antibodies against NFATc1 and β-actin for 1 h. After washing, horseradish peroxidase-conjugated secondary antibodies were added, and the membranes were incubated for 1 h at room temperature. After washing, the hybridized bands were detected using an ECL detection kit (Pierce, Rockford, IL, USA) and Hyperfilm (Agfa, Belgium).

### Statistical Analysis

Statistical analysis was performed using GraphPad Prism (version 5 for Windows; GraphPad Software, San Diego, CA, USA). *P* values were calculated using a two-tailed paired *t*-test. *P*<0.05 was considered statistically significant.

## Results

### *In vivo* Effects of GSPE Treatment

Treatment with GSPE ameliorated arthritic symptoms during the course of disease (Fig. 1A, B). To assess the *in vivo* effect of GSPE on osteoclastogenesis, bone marrow cells from GSPE-treated mice were induced to undergo osteoclast differentiation by culturing them in the presence of M-CSF (10 ng/mL) and RANKL (50 ng/mL). Fewer TRAP^+^ MNCs were formed in the GSPE-treated mice than in the CIA mice (Fig. 1C). Histological sections of the tibiotalar joint of the ankle were stained with TRAP, NFATc1, RANK, 7, Tcf3, and nitrotyrosine, which is an indicator of cell damage, inflammation and NO production. GSPE-treated mice had less severe CIA compared to the saline-treated mice and had features that were similar to those observed in normal mice. Fewer NFATc1, nitrotyrosine, RANK, LRP4, which antagonizes canonical Wnt signaling [28], and Tcf3-positive cells were observed in the joints of GSPE-treated mice compared to saline-treated mice (Fig. 1D).

### The Suppressive Effect of GSPE on Osteoclast Differentiation *in vitro*

To verify the suppressive effect of GSPE on osteoclast differentiation *in vitro*, mouse bone marrow cells were differentiated into osteoclasts in the presence of M-CSF, RANKL, and various concentrations of GSPE (1 or 10 µg/mL). GSPE treatment suppressed osteoclast differentiation *in vitro* in a dose-dependent manner (Fig. 2A). The functional activity of osteoclast was assessed in a bone resorption assay system performed in 24-well OAAS plates coated with a thin film of calcium phosphate. The resorbed area was smaller after treatment with GSPE, and this effect was dose-dependent (Fig. 2B). GSPE did not affect the cytotoxicity of BMMs at concentrations up to 20 µg/mL, and these data suggested that the suppressive effect of GSPE on osteoclast development was not caused by cell death (Fig. 2C). To determine whether GSPE regulated the mRNA levels of molecules known to be strongly expressed in osteoclasts, the relative mRNA expression levels of MMP9, cathepsin K, TRAP, carbonic anhydrase II, integrin-β, and calcitonin receptor were measured using quantitative real-time PCR. The addition of GSPE to the cultures inhibited the induction of MMP9, cathepsin K, TRAP, carbonic anhydrase II, integrin-β, and calcitonin receptor almost completely (Fig. 2D). Osteoclast-related molecules, such as TRAP, calcitonin receptor, cathepsin K, and MMP9, were induced specifically by RANKL. It has been reported that NFATc1 plays a critical role as a master switch in the terminal differentiation of osteoclasts by functioning downstream of RANKL [29]. To investigate the role of NFATc1 in GSPE-treated osteoclasts, its relative mRNA expression level was measured using real-time PCR. As shown in Fig. 2E, 10 µg/mL GSPE was sufficient to markedly suppress NFATc1 mRNA levels. Western blot analysis also showed that the expression of NFATc1 was significantly downregulated in osteoclasts in the presence of GSPE. NFATc1 was intensely expressed in areas of bone destruction in the joints of CIA mice when compared to the GSPE-treated mice (Fig. 1D).

### The Effect of GSPE on Osteoblast Differentiation

Rcan3, a calcineurin inhibitor that downregulates NFAT-dependent cytokine gene expression [30], and Sox6, a potent enhancer of chondroblast function [31] were detected more prominently in the joints of GSPE-treated mice (Fig. 3). To examine the effect of GSPE on osteoblast differentiation, we cultured primary murine osteoblasts and MC3T3-E1, an osteoblast cell line, in the presence of β-glycerophosphate, ascorbic acid, and BMP-2. As shown in Fig. 4A and 4C, treatment of osteoblasts with GSPE significantly increased ALP activity and mineralization compared to the untreated cells. To investigate the regulatory mechanism through which GSPE positively controlled osteoblast differentiation, the role of GSPE in the expression of osteoblast differentiation-related genes, such as ALP, Runx2, and osterix was examined (Fig. 4B, D). GSPE increased the expression of these genes compared to the GSPE-untreated condition. GSPE also inhibited the induction of iNOS in a dose-dependent manner (Fig. 4E).

### The Effect of GSPE on Rheumatoid Arthritis Synovial Fibroblasts

Treatment with inflammatory cytokines, such as IL-1β and TNF-α, induces RANKL expression in RA synoviocytes [32]. To investigate whether GSPE could regulate the mRNA expression of RANKL on fibroblasts from RA patients, RA FLS with IL-1β or TNF-α in the presence of GSPE was cultured. GSPE treatment suppressed the mRNA expression of RANKL in FLS (Fig. 5A). RANKL and nitrotyrosine were highly expressed in synovial tissue from RA patients, whereas there were few positive cells observed in osteoarthritis (OA) synovial tissue (Fig. 5B).

### Suppressive Effect of GSPE on Human Monocyte-derived Osteoclasts

To assess whether GSPE suppressed human osteoclast differentiation, human PBMC-derived monocytes were cultured with GSPE in the presence of M-CSF and RANKL, and TRAP^+^ cells were detected by TRAP staining. GSPE inhibited the differentiation of TRAP^+^ MNCs in a dose-dependent manner, similarly to what was observed in mice (Fig. 6A). The mRNA expression levels of TRAP, cathepsin K, MMP9, and calcitonin receptor were reduced after GSPE treatment (Fig. 6B). LRP4 expression was stronger in the RA synovium compared to the OA synovium (Fig. 6C). Oxidative stress plays an important role in osteoclast differentiation and function. RANKL-induced intracellular ROS production stimulates RANKL signaling pathways and thus, promoting osteoclast differentiation and function [33], [34]. To identify the inhibitory role of GSPE in ROS signaling, human PBMC-derived monocytes were cultured with GSPE in the presence of M-CSF, RANKL, and H_2_O_2_, and TRAP^+^ cells were detected by TRAP staining. Treatment with H_2_O_2_ significantly promoted osteoclast differentiation compared to the M-CSF plus RANKL-treated condition whereas GSPE suppressed the formation of osteoclast (Fig. 6D).

## Discussion

GSPE, an antioxidant obtained from the seeds of *Vitis vinifera*, scavenges the free radicals that are required for calcium absorption [18], and has been documented to have beneficial biological properties against various pathological diseases [14], [15], [16]. Based on this evidence, we reasoned that GSPE might attenuate the severity of RA. The present study showed that GSPE effects on bone invasion in arthritis by simultaneously suppressing osteoclast differentiation and promoting osteoblast differentiation.

We recently reported on the antiarthritic effect of GSPE by reducing the production of type-II collagen-specific IgG2a and inflammatory cytokines, such as TNF-α and IL-17 [35]. Our group also investigated the role of GSPE in the regulation of IL-17-producing T cells and regulatory T cells, which play a significant role in the innate immune response. We found that GSPE has a dual effect by reducing IL-17 production and increasing forkhead box P3 expression [36]. Several inflammatory cytokines and pathogenic T cells, such as IL-17-producing T cells, are implicated in joint destruction [23], [37], a major symptom of RA. These data suggest that GSPE may inhibit bone destruction caused by the imbalance between osteoclasts and osteoblasts. Our previous study showed that GSPE reduced the production of H_2_O_2_ via anti-CD3 antibody treatment of CD4^+^ T cells [35]. These observations are similar to a previous report, where GSPE inhibited expression of the cell-surface receptor for advanced glycation end product in endothelial cells by suppressing ROS generation [38]. High concentrations of ROS may cause oxidative stress, leading to the damage of cellular macromolecules, which may interfere with various cellular functions. In bone, osteoclasts have NADPH oxidase components. A finding by Steinbeck MJ et al. supported the involvement of NADPH oxidase and ROS in the process of bone resorption by osteoclasts [39]. In addition, RANKL, induced the generation of ROS in bone marrow cells and osteoclasts [34]. These studies indicated that mature osteoclasts produce ROS, perhaps through activated NADPH oxidase, and that ROS may in turn increase the formation and activation of osteoclasts. Our study revealed that GSPE inhibited osteoclastogenesis induced by H_2_O_2_ treatment, implicating its antioxidative effect in inhibiting osteoclastogenesis.

In this study, GSPE attenuated the severity of the disease *in vivo* in the CIA mouse model and decreased the number of TRAP^+^ MNCs in the GSPE-treated group compared to the saline-treated group. We found a limited spectrum of antiresorptive and antiarthritic effects, although GSPE may have a more diverse immunological effect *in vivo*. Future studies are needed to elucidate its immunological roles. We showed that GSPE treatment suppressed osteoclast differentiation and activity in a dose-dependent manner in osteoclasts induced by mouse bone marrow cells and human monocytes. In addition, GSPE inhibited the expression of osteoclast-related genes, such as MMP9, cathepsin K, calcitonin receptor, RANK, TRAP, and NFATc1. NFATc1 is induced by RANKL/RANK-associated signaling pathways and is considered to be a master regulator of the induction of osteoclast-specific genes, such as TRAP and calcitonin receptor [29]. Previous enzymatic studies demonstrated that proanthocyanidin inhibited proteolytic enzymes, which play an essential role in the initial process of bone resorption [40], [41]; this seems to support the suppressive effects of GSPE in osteoclasts. Our experiments with synovial fibroblasts from RA patients demonstrated stimulation by inflammatory cytokines, which contributed to the increased expression of RANKL in RA synoviocytes. This suggests that GSPE has the potential to downregulate RANKL. Our results imply that GSPE may be a potent suppressor of osteoclast differentiation.

Interestingly, the antiarthritic effects of GSPE were not limited to the inhibition of osteoclasts. Our data demonstrated that GSPE also functions to increase osteoblast differentiation. Concomitantly, the expression of osteoblast markers was upregulated in primary osteoblasts. In support of this, GSPE was previously reported to induce additional bone formation in rat mandibular condyles [42]. The effectiveness of therapies with a dual mode of action similar to that of GSPE in the treatment of bone destruction was reported for osteoporosis [43]. Natural compounds, such as GSPE, may provide an adequate and safe alternative or complimentary treatment from the traditional use of drugs for the treatment of bone destruction in inflammatory conditions such as RA.

In this study, we demonstrated that GSPE strongly inhibited osteoclast differentiation and reduced osteoclast activity. Furthermore, GSPE stimulated bone formation through its positive action on osteoblast differentiation. The balance between osteoblast and osteoclast activity is important for maintaining bone quality. Our results suggest that GSPE regulates bone homeostasis via dual actions and may be a potential therapeutic agent for the treatment of bone destruction in RA.
